# Nicotine Is Associated with Improved Histological and Biochemical Indices of Oral Ulcer Repair in an Acetic Acid-Induced Rat Model

**DOI:** 10.3390/medicina62050900

**Published:** 2026-05-07

**Authors:** İrem Hengirmen Acu, Oytun Erbaş

**Affiliations:** 1 Department of Dermatology and Venereology, Faculty of Medicine, Ufuk University, 06510 Ankara, Türkiye; 2 Faculty of Medicine, BAMER, Biruni University, 34015 Istanbul, Türkiye; oytunerbas2012@gmail.com

**Keywords:** oral ulcer, nicotine, oxidative stress, inflammation, VEGF-A, EGFR

## Abstract

*Background and Objectives:* This study aimed to evaluate the association between systemic nicotine administration and histological and biochemical repair endpoints in an acetic acid-induced rat oral ulcer model. *Materials and Methods:* Thirty-six male Wistar rats were assigned to control, oral ulcer + saline, and oral ulcer + nicotine (1 mg/kg/day, s.c.) groups. Oral ulcers were induced with 70% acetic acid. After 15 days, buccal mucosa and plasma samples were collected for histopathological and biochemical analyses. Epithelial thickness and fibrosis were assessed histologically, while malondialdehyde (MDA), tumor necrosis factor-α (TNF-α), vascular endothelial growth factor-A (VEGF-A), and epidermal growth factor receptor (EGFR) were quantified. *Results:* Relative to controls, ulcer induction was associated with reduced epithelial thickness and increased fibrosis, MDA, and TNF-α levels. Compared with the oral ulcer + saline group, the nicotine-treated group showed greater epithelial thickness, lower fibrosis, lower MDA and TNF-α levels, and higher VEGF-A and EGFR levels at the study endpoint. No significant difference in VEGF-A was observed between the control and oral ulcer + saline groups. *Conclusions:* In this acetic acid-induced rat model, systemic nicotine administration was associated with improved endpoint histological and biochemical indices of oral ulcer repair. Because macroscopic wound closure, dose–response relationships, route comparisons, and direct mechanistic experiments were not included, these findings should be interpreted as preliminary preclinical associations rather than evidence of a direct causal effect of nicotine on wound healing.

## 1. Introduction

Oral mucosal ulceration is a common clinical condition characterized by disruption of epithelial integrity and underlying connective tissue involvement, typically associated with pain and inflammation. These lesions may result from diverse etiological factors, including local trauma, infections, immune-mediated disorders, systemic diseases, and certain medications such as nicorandil, captopril, and nonsteroidal anti-inflammatory drugs [1,2,3]. Although many ulcers are self-limiting, those persisting beyond two weeks are considered chronic and may require targeted therapeutic intervention [4]. Current treatment options, including corticosteroids and immunomodulators, are widely used but may be limited by inconsistent efficacy and adverse effects [5].

Oral wound healing is a tightly regulated process involving hemostasis, inflammation, proliferation, and remodeling [6]. Compared to skin, oral mucosa generally heals more rapidly due to its saliva-rich environment and rapid epithelial turnover; however, persistent inflammation or dysregulated remodeling may lead to fibrosis and impaired function [7,8]. Therefore, correlating molecular pathways with histological outcomes such as epithelial thickness and fibrosis is critical for understanding repair mechanisms.

Oxidative stress is a key contributor to ulcer pathogenesis, promoting lipid peroxidation and sustaining inflammatory signaling. Malondialdehyde (MDA) is a reliable indicator of oxidative injury, and its elevation, along with increased TNF-α levels, has been associated with delayed mucosal healing [9,10,11]. Effective tissue repair depends on regulated epithelial proliferation and migration, processes regulated in part by epidermal growth factor receptor (EGFR) signaling, with impaired EGFR activity leading to delayed re-epithelialization [12].

Nicotine, a major component of tobacco, exhibits complex, dose-dependent effects on inflammation and tissue repair. Acting through nicotinic acetylcholine receptors, nicotine can exert both pro- and anti-inflammatory effects depending on the biological context [13]. Activation of the α7 nicotinic receptor mediates anti-inflammatory responses by suppressing macrophage cytokine release and inhibiting NF-κB signaling [14]. Experimental studies demonstrate that nicotine administration at 1 mg/kg/day reduces pro-inflammatory cytokines. However, its effects on angiogenesis remain controversial, with reports of both increased VEGF expression and impaired vascular density [15,16,17]. Given this duality, further investigation is warranted.

The acetic acid-induced oral ulcer model provides a reproducible experimental platform for evaluating molecular and histopathological aspects of mucosal repair [18,19]. In this study, we aimed to assess the effects of nicotine on oxidative stress (MDA), inflammation (TNF-α), angiogenesis (VEGF-A), and epithelial signaling (EGFR), as well as histopathological parameters, including epithelial thickness and fibrosis. We hypothesized that nicotine modulates oxidative and inflammatory pathways, thereby influencing oral wound healing outcomes.

## 2. Materials and Methods

### 2.1. Animals

A total of 36 adult male Wistar albino rats (200–250 g) were included in this experimental study. All experimental procedures were conducted in strict accordance with the Guide for the Care and Use of Laboratory Animals published by the National Institutes of Health (USA). Ethical approval was obtained from the Animal Experiments Local Ethics Committee of Science University (Approval No.: 26025610; Date: 18 December 2023).

The animals were obtained from the Experimental Animal Research Center of Science University. Throughout the study, rats were housed in pairs in stainless-steel cages under controlled environmental conditions (ambient temperature: 22 ± 2 °C; 12 h light/dark cycle) and had free access to standard laboratory chow and water.

To minimize biological variability, animals were selected to ensure comparable age and body weight distributions across groups. In addition, all environmental parameters, including housing conditions, dietary content, and circadian rhythm, were standardized and maintained consistently throughout the experimental period.

### 2.2. Experimental Protocol

#### 2.2.1. Induction of Oral Ulcer Model

Oral ulceration was induced in 24 rats by applying 70% acetic acid (AA) (Merck Sigma-Aldrich, St. Louis, MO, USA) to the left buccal mucosa. The procedure was performed under ketamine anesthesia (50 mg/kg, intraperitoneal).

Before application, the oral mucosa was gently dried to ensure uniform exposure. A cotton-tipped applicator (5 mm in diameter) was saturated with 70% acetic acid for 3–5 s and then applied to the buccal mucosa for 1 min to produce a standardized ulcer lesion. The remaining 12 rats did not undergo ulcer induction and were used as the normal control group. To ensure consistency in handling and injection-related stress, all groups received daily subcutaneous injections, with saline administered to the control and ulcer groups.

#### 2.2.2. Experimental Groups

Animals were randomly allocated into three experimental groups (n = 12 per group) as follows:

Group 1 Control (n = 12):

No oral ulcer induction; animals received 0.9% NaCl (1 mL/kg/day, subcutaneous).

Group 2 Oral ulcer + saline (n = 12):

An oral ulcer was induced using 70% acetic acid; animals received 0.9% NaCl (1 mL/kg/day, subcutaneous).

Group 3 Oral ulcer + nicotine (n = 12):

An oral ulcer was induced using 70% acetic acid; animals received nicotine (1 mg/kg/day, subcutaneous).

### 2.3. Treatment Protocol, Sample Collection

All treatments were administered once daily for 15 consecutive days, and the route of administration was kept consistent across treatment groups via subcutaneous injection. At the end of the experimental period, all animals were euthanized under deep anesthesia using ketamine (75 mg/kg) and xylazine (15 mg/kg). Blood samples were collected by cardiac puncture for subsequent biochemical analyses. A standardized 10 mm section of the left buccal mucosa was carefully excised and processed for both histopathological and biochemical evaluations.

To ensure methodological rigor and reduce potential sources of bias, animals were randomly assigned to the experimental groups, and histopathological assessments were performed in a blinded manner. In addition, all ELISA and biochemical measurements were conducted in duplicate to enhance the reliability and reproducibility of the results.

### 2.4. Histopathological Evaluation of Buccal Mucosa

Formalin-fixed buccal mucosal tissues were embedded in paraffin, sectioned at 4 μm thickness, and stained with hematoxylin and eosin (H&E). Histological images were captured using an Olympus BX51 microscope equipped with an Olympus C-5050 digital camera (Olympus Corp., Tokyo, Japan).

Histopathological evaluation focused on epithelial thickness and fibrosis. Epithelial thickness was quantitatively measured in micrometers (µm) at multiple randomly selected fields per section using image analysis software. The mean epithelial thickness of the control group was set to 100%, and individual values in other groups were expressed as percentages relative to the control mean [(measured value/control mean) × 100] to reduce inter-sample variability and facilitate comparisons across groups. Fibrosis was evaluated semi-quantitatively by estimating the proportion of fibrotic area within the mucosal tissue and was expressed as a percentage of the total examined area.

### 2.5. Biochemical Analysis of Buccal Mucosa (TNF-α, VEGF-A, EGFR)

Immediately after euthanasia, buccal mucosal tissues were rapidly excised and stored at −20 °C until biochemical analysis. For tissue preparation, samples were homogenized in ice-cold phosphate-buffered saline (PBS, pH 7.4) at a 1:5 (*w*/*v*) ratio using a glass homogenizer. The homogenates were then centrifuged at 5000× *g* for 15 min, and the resulting supernatants were collected for further analysis.

Total protein concentrations in the tissue homogenates were determined using the Bradford assay with bovine serum albumin as the standard. The levels of TNF-α, VEGF-A, and EGFR in the buccal mucosa supernatants were quantified using commercially available rat-specific enzyme-linked immunosorbent assay (ELISA) kits, according to the manufacturers’ protocols. All samples were analyzed in duplicate to ensure reliability. Absorbance values were measured using a microplate reader (Multiskan Go, Thermo Fisher Scientific, Newington, NH, USA).

### 2.6. Measurement of Plasma Lipid Peroxidation

Plasma lipid peroxidation was evaluated by determining malondialdehyde (MDA) levels using the thiobarbituric acid reactive substances (TBARS) method. Briefly, plasma samples were mixed with trichloroacetic acid and the thiobarbituric acid reagent and then incubated at 100 °C for 60 min to allow formation of the MDA–TBA adduct.

**Figure 1:**
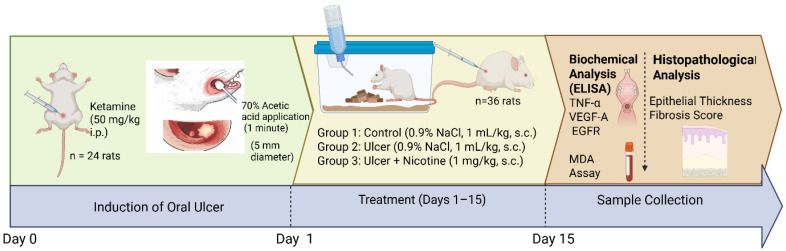
Experimental design of the acetic acid-induced oral ulcer model and treatment protocol. Oral ulceration was induced in rats by applying 70% acetic acid to the left buccal mucosa under ketamine anesthesia (50 mg/kg, intraperitoneal). A cotton-tipped applicator (5 mm diameter) soaked in acetic acid was applied for 1 min to create a standardized ulcer lesion. Following ulcer induction (day 0), animals were randomly assigned to three groups: control (no ulcer induction, 0.9% NaCl, 1 mL/kg, s.c.), oral ulcer + saline (0.9% NaCl, 1 mL/kg, s.c.), and oral ulcer + nicotine (1 mg/kg, s.c.). Treatments were administered once daily for 15 days (days 1–15). At the end of the experimental period (day 15), samples were collected for biochemical analyses, including ELISA-based measurement of TNF-α, VEGF-A, and EGFR levels, as well as lipid peroxidation assessment by MDA assay. Histopathological evaluation was performed to assess epithelial thickness and fibrosis. The figure summarizes the experimental timeline, including ulcer induction, treatment, and sample collection.

After incubation, samples were rapidly cooled on ice and centrifuged at 3000 rpm for 20 min to obtain a clear supernatant. The absorbance of the supernatant was measured spectrophotometrically at 535 nm. MDA concentrations were calculated using a standard calibration curve generated with tetraethoxypropane and expressed as nmol/L (Figure 1).

### 2.7. Statistical Analysis

All statistical analyses were performed using SPSS software (version 15.0 for Windows; SPSS Inc., Chicago, IL, USA). Graphs were generated using GraphPad Prism software (version 9.0; GraphPad Software Inc., San Diego, CA, USA). Data distribution was assessed using the Shapiro–Wilk test, and all variables were found to be approximately normally distributed across groups (*p* > 0.05). Given the equal group sizes, one-way analysis of variance (ANOVA) followed by Tukey’s post hoc test was used for comparisons. A multivariate analysis of variance (MANOVA) was also conducted to evaluate the overall effect of group allocation on the combined histological and biochemical outcome variables, with Pillai’s Trace as the multivariate test statistic. In addition to *p*-values, F-statistics, 95% confidence intervals, and partial eta-squared (ηp^2^) effect sizes are reported as part of the relevant analyses. Results are presented as mean ± standard error of the mean (SEM), and a *p*-value of less than 0.05 was considered statistically significant.

## 3. Results

A multivariate analysis of variance (MANOVA) revealed a significant overall effect of group on the combined outcome variables (Pillai’s Trace = 1.523, F(12,58) = 15.448, *p* < 0.001, ηp^2^ = 0.76). Subsequent univariate analyses were performed to determine the contribution of individual parameters to this overall effect.

### 3.1. Histopathological Analysis

#### 3.1.1. Histopathological Examination in Figure 2

**Figure 2:**
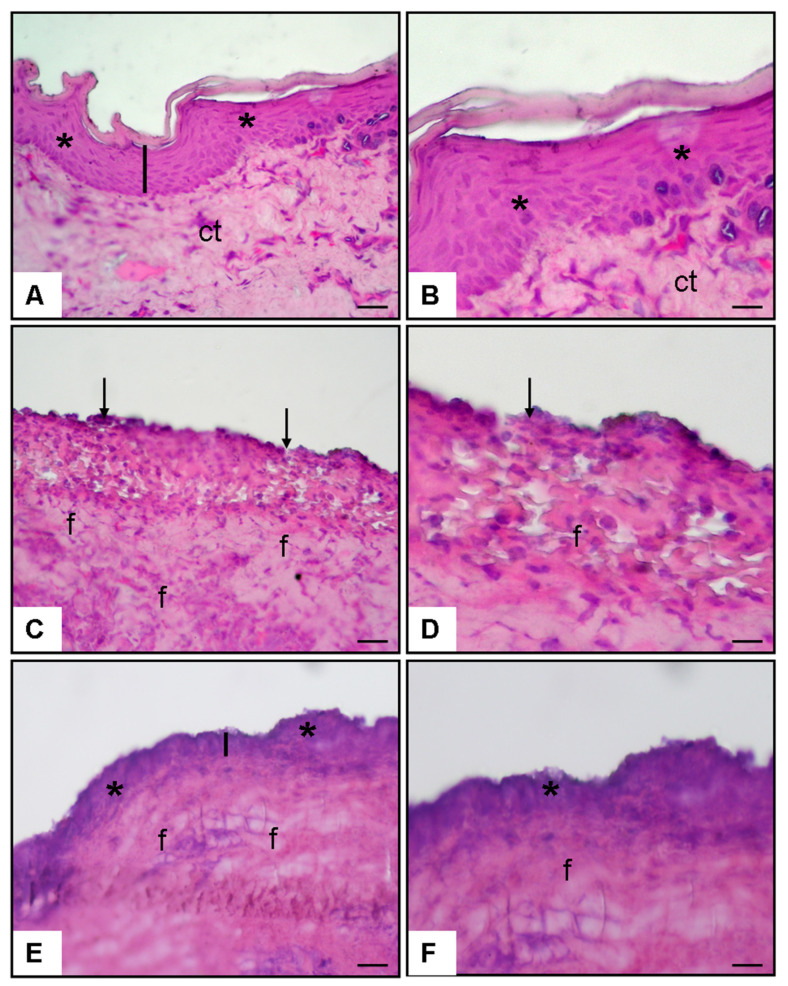
Histopathological findings at ×20 and ×40 magnifications (hematoxylin and eosin staining): (**A**,**B**) Normal control group buccal mucosa showing intact epithelium (asterisk) and normal epithelial thickness (line). (**C**,**D**) Oral ulcer + 0.9% NaCl group demonstrating disrupted epithelium (arrows) and increased fibrosis (f). (**E**,**F**) Oral ulcer + nicotine group showing re-epithelialization (asterisk) and partial restoration of epithelial thickness (line).

Histopathological examination revealed a well-organized epithelial structure with preserved thickness in the control group. In contrast, the oral ulcer group exhibited marked epithelial disruption, surface erosion, and increased subepithelial fibrosis. Notably, nicotine treatment promoted re-epithelialization and partially restored epithelial thickness, accompanied by a reduction in fibrotic changes compared to the ulcer group (Figure 2).

#### 3.1.2. Epithelial Thickness

**Figure 3:**
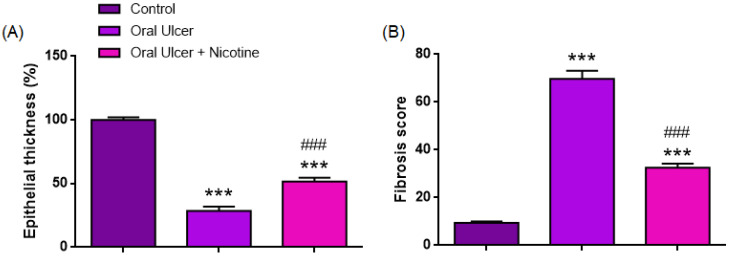
Quantitative analysis of epithelial thickness and fibrosis score: (**A**) Epithelial thickness (%) and (**B**) fibrosis score across experimental groups. The oral ulcer group showed a marked reduction in epithelial thickness and a significant increase in fibrosis compared to the control group. Nicotine treatment significantly increased epithelial thickness and reduced fibrosis compared to the oral ulcer group, although values did not fully return to control levels. Data are presented as mean ± SEM (n = 12 per group). Statistical analysis was performed using one-way ANOVA followed by Tukey’s multiple comparisons test. *** *p* < 0.001 vs. control group; ^###^ *p* < 0.001 vs. oral ulcer group.

As shown in Table 1 and illustrated in Figure 3, epithelial thickness (%) differed significantly among the groups (one-way ANOVA, F(2,33) = 169.7, *p* < 0.0001, ηp^2^ = 0.91). The mean epithelial thickness was markedly reduced in the oral ulcer group (28.6 ± 3.38%) compared with the control group (100.0 ± 2.03%, *p* < 0.0001). In contrast, the oral ulcer + nicotine group demonstrated a significant increase in epithelial thickness (51.7 ± 2.81%) compared with the ulcer group (*p* < 0.0001). However, values remained significantly lower than those of the control group (*p* < 0.0001). Epithelial thickness was significantly decreased in the ulcer group compared with the control (mean difference: 71.42%; 95% CI: 61.71–81.12; *p* < 0.0001). Similarly, the oral ulcer + nicotine group exhibited significantly lower epithelial thickness than the control group (mean difference: 48.26%, 95% CI: 38.56–57.97, *p* < 0.0001). Moreover, epithelial thickness was significantly higher in the oral ulcer + nicotine group compared to the ulcer group (mean difference: −23.15%, 95% CI: −32.86 to −13.45, *p* < 0.0001).

#### 3.1.3. Fibrosis Score

As summarized in Table 1 and depicted in Figure 3, fibrosis scores differed significantly among the groups (one-way ANOVA, F(2,33) = 185.2, *p* < 0.0001, ηp^2^ = 0.92). The mean fibrosis score was markedly increased in the oral ulcer group (69.69 ± 3.38) compared with the control group (9.46 ± 0.59, *p* < 0.0001). In contrast, the oral ulcer + nicotine group demonstrated a significant reduction in fibrosis score (32.48 ± 1.78) compared with the ulcer group (*p* < 0.0001). However, values remained significantly higher than those of the control group (*p* < 0.0001). Post hoc Tukey analysis confirmed that all pairwise comparisons were statistically significant. Fibrosis scores were significantly higher in the ulcer group than in the control group (mean difference: −60.23, 95% CI: −67.98 to −52.48, *p* < 0.0001). Similarly, fibrosis scores in the oral ulcer + nicotine group remained significantly higher than those in the control group (mean difference: −23.02, 95% CI: −30.77 to −15.27, *p* < 0.0001). Notably, fibrosis scores were significantly reduced in the oral ulcer + nicotine group compared to the ulcer group (mean difference: 37.21, 95% CI: 29.46–44.96, *p* < 0.0001).

### 3.2. Biochemical Analysis

#### 3.2.1. Plasma MDA Level (nM)

**Figure 4:**
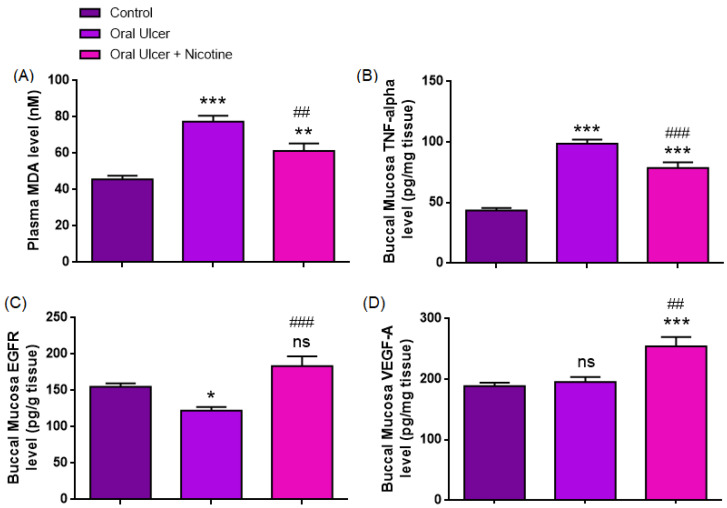
Biochemical parameters in buccal mucosa and plasma across experimental groups: (**A**) Plasma MDA levels (nM), (**B**) buccal mucosa TNF-α levels (pg/mg tissue), (**C**) buccal mucosa EGFR levels (pg/g tissue), and (**D**) buccal mucosa VEGF-A levels (pg/mg tissue). The oral ulcer group showed significantly higher MDA and TNF-α levels and lower EGFR levels than the control group. Nicotine treatment significantly reduced MDA and TNF-α levels and increased EGFR and VEGF-A levels compared to the oral ulcer group. No significant difference was observed between the control and oral ulcer groups in VEGF-A levels. Data are presented as mean ± SEM (n = 12 per group). Statistical analysis was performed using one-way ANOVA followed by Tukey’s multiple comparisons test. * *p* < 0.05, ** *p* < 0.01, *** *p* < 0.001 vs. control group; , ^##^ *p* < 0.01, ^###^ *p* < 0.001 vs. oral ulcer group; ^ns^: not significant.

As presented in Table 1 and Figure 4, plasma MDA levels differed significantly among the groups (one-way ANOVA, F(2,33) = 23.44, *p* < 0.0001, ηp^2^ = 0.59). The mean MDA level was significantly increased in the oral ulcer group (77.45 ± 3.29 nM) compared with the control group (45.63 ± 2.03 nM, *p* < 0.0001). In contrast, the oral ulcer + nicotine group exhibited a significant reduction in MDA levels (61.29 ± 4.17 nM) compared with the ulcer group (*p* = 0.0040). However, values remained significantly higher than those of the control group (*p* = 0.0053). Post hoc Tukey analysis confirmed that MDA levels were significantly elevated in the ulcer group compared with the control group (mean difference: −31.83 nM, 95% CI: −43.23 to −20.42, *p* < 0.0001). Similarly, MDA levels in the oral ulcer + nicotine group remained significantly higher than those in the control group (mean difference: −15.67 nM, 95% CI: −27.07 to −4.26, *p* = 0.0053). Notably, MDA levels were significantly reduced in the oral ulcer + nicotine group compared to the ulcer group (mean difference: 16.16 nM, 95% CI: 4.75–27.57, *p* = 0.0040).

#### 3.2.2. Buccal Mucosa TNF-α Level (pg/mg Tissue)

The findings presented in Table 1 and Figure 4 demonstrate that buccal mucosal TNF-α levels differed significantly among the groups (one-way ANOVA, F(2,33) = 64.61, *p* < 0.0001, ηp^2^ = 0.80). The mean TNF-α level was markedly elevated in the oral ulcer group (98.69 ± 3.42 pg/mg tissue) compared with the control group (43.61 ± 1.93 pg/mg tissue, *p* < 0.0001). In the oral ulcer + nicotine group, TNF-α levels (78.73 ± 4.55 pg/mg tissue) were significantly reduced compared with the ulcer group (*p* = 0.0008). However, they remained significantly higher than those observed in the control group (*p* < 0.0001).

The TNF-α levels were significantly increased in the ulcer group compared to the control (mean difference: −55.08 pg/mg tissue, 95% CI: −67.12 to −43.04, *p* < 0.0001). Similarly, TNF-α levels in the oral ulcer + nicotine group remained significantly higher than those in the control group (mean difference: −35.13 pg/mg tissue, 95% CI: −47.16 to −23.09, *p* < 0.0001). Notably, TNF-α levels were significantly decreased in the oral ulcer + nicotine group compared to the ulcer group (mean difference: 19.96 pg/mg tissue, 95% CI: 7.92–32.00, *p* = 0.0008).

#### 3.2.3. Buccal Mucosa EGFR Level (pg/g Tissue)

The findings presented in Table 1 and Figure 4 indicate that buccal mucosal EGFR levels differed significantly among the groups (one-way ANOVA, F(2,33) = 12.76, *p* < 0.0001, ηp^2^ = 0.44). The mean EGFR level was significantly decreased in the oral ulcer group (122.34 ± 5.04 pg/g tissue) compared with the control group (155.07 ± 4.72 pg/g tissue, *p* = 0.0290). In contrast, the oral ulcer + nicotine group exhibited higher EGFR levels (183.69 ± 13.19 pg/g tissue) compared with the ulcer group (*p* < 0.0001). However, no statistically significant difference was observed between the control and oral ulcer + nicotine groups (*p* = 0.0620). A significant reduction in EGFR levels was observed in the ulcer group compared to the control group (mean difference: 32.72, 95% CI: 2.90–62.55, *p* = 0.0290). While EGFR levels tended to be higher in the oral ulcer + nicotine group than in the control group, this difference did not reach statistical significance (mean difference: −28.63, 95% CI: −58.45 to 1.99, *p* = 0.0620). Notably, EGFR levels were significantly increased in the oral ulcer + nicotine group compared to the ulcer group (mean difference: −61.35, 95% CI: −91.17 to −31.52, *p* < 0.0001).

#### 3.2.4. Buccal Mucosa VEGF-A Level (pg/mg Tissue)

The results presented in Table 1 and Figure 4 indicate that buccal mucosal VEGF-A levels differed significantly among the groups (one-way ANOVA, F(2,33) = 11.63, *p* = 0.0002, ηp^2^ = 0.41). No statistically significant difference was observed between the control and oral ulcer groups (188.85 ± 5.51 vs. 195.38 ± 8.25 pg/mg tissue, *p* = 0.9007). In contrast, VEGF-A levels were significantly increased in the oral ulcer + nicotine group (254.30 ± 15.39 pg/mg tissue) compared with both the control group (*p* = 0.0003) and the ulcer group (*p* = 0.0011). No significant difference in VEGF-A levels was observed between the control and ulcer groups (mean difference: −6.53, 95% CI: −43.21 to 30.16, *p* = 0.9007). However, VEGF-A levels were significantly higher in the oral ulcer + nicotine group compared to the control (mean difference: −65.45, 95% CI: −102.1 to −28.76, *p* = 0.0003) and the ulcer group (mean difference: −58.93, 95% CI: −95.61 to −22.24, *p* = 0.0011).

## 4. Discussion

The present findings indicate that, in this endpoint rat model, systemic nicotine administration was associated with differences in histological and biochemical indices of oral ulcer repair. Relative to the untreated ulcer group, nicotine exposure was associated with greater epithelial thickness, lower fibrosis, reduced TNF-α and MDA levels, and higher EGFR and VEGF-A levels at the study endpoint. These group-level differences across both histological and biochemical parameters further support the association between experimental conditions and oral mucosal injury and repair. However, the present design does not allow conclusions regarding direct causality or underlying mechanisms; therefore, these findings should be interpreted as associations within a controlled preclinical model.

Epithelial thickness is a key parameter reflecting keratinocyte survival, proliferation, and migration. Oral mucosa typically heals rapidly with minimal scarring; however, this advantage is diminished in the presence of persistent inflammatory conditions [20,21]. The pronounced epithelial thinning in the ulcer group reflects the destructive phase of chemical injury. In contrast, the increase in epithelial thickness observed following nicotine treatment may reflect enhanced re-epithelialization, potentially associated with reduced inflammatory burden and epithelial growth-related responses.

Fibrosis showed an inverse pattern. Ulcer induction increased the fibrosis score, whereas nicotine treatment was associated with lower fibrosis values. In oral wound models, excessive fibrosis is typically linked to prolonged inflammation and dysregulated extracellular matrix (ECM) deposition rather than effective healing [8]. Accordingly, the reduction in fibrosis, together with increased epithelial thickness, may reflect a shift toward more organized tissue remodeling. This finding is particularly relevant to the oral mucosa, which normally heals with minimal scarring compared with skin [20,21]. Although nicotine has been associated with fibrogenic processes in other tissues, suggesting context-dependent effects [22], the present results suggest that, under these experimental conditions, nicotine exposure may be associated with reduced fibrosis alongside improved structural features.

Oral mucosal repair depends on tightly regulated interactions among inflammation, oxidative balance, epithelial migration and proliferation, angiogenic support, and extracellular matrix remodeling [23]. Acetic acid-induced injury is widely used due to its reproducibility and predictable healing course [24]. In this model, epithelial thickness and fibrosis reflect structural aspects of tissue repair, while TNF-α and malondialdehyde (MDA) indicate inflammatory and oxidative status [25,26,27,28]. These processes are closely interconnected in oral ulcer pathology [9,29]. The pattern observed in the present study is consistent with previous reports describing anti-inflammatory effects of nicotine mediated through the cholinergic anti-inflammatory pathway, in which α7 nicotinic acetylcholine receptor signaling suppresses macrophage cytokine production and inhibits NF-κB activity [14]. Previous studies have also shown that nicotine at comparable doses reduces pro-inflammatory cytokines [15]. Accordingly, the observed changes in TNF-α may be associated with a neuroimmune response that could contribute to the overall histological pattern.

In the present study, nicotine was associated with higher buccal mucosal EGFR levels compared to the ulcer group. These findings are consistent with the established role of EGFR in regulating keratinocyte proliferation and migration, which are essential for re-epithelialization and wound closure [30,31]. In response to tissue injury, EGFR activation has been reported to involve ADAM17-mediated shedding of ligands such as HB-EGF, which promotes epithelial migration [32,33]. Similarly, TNF-α is a key mediator of tissue injury and has been reported to impair epithelial repair by inhibiting EGFR activation [34]. Accordingly, the observed changes in EGFR and TNF-α may be associated with epithelial and inflammatory responses related to tissue repair. This pattern is also in line with reports describing anti-inflammatory effects of nicotine mediated through the cholinergic anti-inflammatory pathway, in which α7 nicotinic acetylcholine receptor signaling suppresses pro-inflammatory cytokine production [14].

Similarly, nicotine was associated with higher VEGF-A levels in the present study. VEGF is a key mediator of angiogenesis and plays an important role in granulation tissue formation and epithelial repair [35]. Previous studies have shown that nicotine can upregulate VEGF expression through HIF-1α-dependent and nicotinic receptor-mediated pathways [16,36,37]. In addition, VEGF-A has been linked to epithelial repair processes, including potential interactions with EGFR-related pathways [38]. However, the present study did not directly assess angiogenesis or vascular structure; therefore, no definitive conclusions regarding angiogenic activity can be drawn. Accordingly, the observed increase in VEGF-A should be interpreted as an association rather than as direct evidence of enhanced angiogenesis or coordinated epithelial–vascular regulation.

A more cautious interpretation is also warranted because nicotine has context-dependent biological effects. Recent reviews describe both anti-inflammatory and pro-inflammatory actions of nicotine, with the direction and magnitude of the effects varying according to dose, duration, route of administration, disease model, and tissue context [13]. Likewise, the broader wound-healing literature is mixed. Nicotine accelerated wound closure and histological healing in diabetic mice in one primary study. Yet, other studies have linked vaping or nicotine exposure to decreased VEGF expression, reduced microvessel density, and poorer wound healing in rats [17,37]. In oral surgical and periodontal settings, tobacco-related vasoconstriction, reduced tissue perfusion, and impaired healing responses remain important concerns, and nicotine has also been implicated in fibrogenic and pathological angiogenic pathways [22,39,40]. For this reason, the present findings should be understood as model-specific rather than universally beneficial.

Taken together, the present findings suggest that nicotine is associated with concurrent changes in inflammatory, oxidative, and repair-related parameters. The observed reductions in TNF-α and MDA, together with higher VEGF-A and EGFR levels, may reflect a shift in the local tissue environment toward conditions more compatible with epithelial repair. However, these observations do not establish a direct mechanistic effect. In addition, although higher VEGF-A levels may be consistent with enhanced wound support, nicotine-related angiogenic responses are known to be context-dependent and may also involve pathological neovascularization [40]. Given nicotine’s pleiotropic biological actions, including its potential involvement in fibrogenic pathways [28], further studies are required before any translational implications can be drawn.

The present study has several limitations. First, only a single nicotine dose, a single systemic route of administration, and a single terminal time point were evaluated; therefore, dose–response relationships, route specificity, and the temporal dynamics of the healing process cannot be determined. Second, serial ulcer-area measurements, clinical wound scoring, photograph-based closure monitoring, and other direct macroscopic assessments of healing were not performed. Accordingly, it remains unclear whether the observed histological and biochemical differences translate into faster visible ulcer closure or clinically meaningful benefit. Future studies should incorporate multiple nicotine doses, comparisons between topical and systemic administration routes, repeated macroscopic assessments, longer follow-up periods, and direct mechanistic investigations targeting nicotinic, EGFR-related, and angiogenic pathways.

Overall, systemic nicotine administration in this acetic acid-induced rat model was associated with differences in endpoint histological and biochemical parameters compared with the ulcer alone. These findings suggest a potential modulatory effect of nicotine on the local wound environment under the specific conditions tested. Still, they should not be interpreted as definitive evidence of direct healing efficacy or clinically significant benefit.

## 5. Conclusions

In conclusion, systemic nicotine administration in this acetic acid-induced rat model was associated with improved endpoint histological and biochemical indices of oral ulcer repair, including greater epithelial thickness, lower fibrosis, reduced TNF-α and MDA levels, and higher EGFR and VEGF-A levels. These findings suggest a potential modulatory effect of nicotine on the wound environment under the specific experimental conditions used here. However, because the study used a single dose, a single systemic route, and a single terminal time point without direct macroscopic healing measurements or mechanistic pathway interrogation, the findings should be interpreted cautiously. They should not be taken as evidence of definitive clinical benefit. Future studies should incorporate dose–response, route-comparison, longitudinal macroscopic, and mechanistic designs.

## Figures and Tables

**Table 1:** Biochemical and histopathological parameters in the experimental groups.

Parameter	Control	Oral Ulcer	Oral Ulcer + Nicotine
Epithelial thickness	100.0 ± 2.03	28.6 ± 3.38 ***	51.7 ± 2.81 ***^,###^
Fibrosis score	9.46 ± 0.59	69.69 ± 3.38 ***	32.48 ± 1.78 ***^,###^
MDA	45.63 ± 2.03	77.45 ± 3.29 ***	61.29 ± 4.17 **^,##^
TNF-α	43.61 ± 1.93	98.69 ± 3.42 ***	78.73 ± 4.55 ***^,###^
EGFR	155.07 ± 4.72	122.34 ± 5.04 *	183.69 ± 13.19 ^ns,###^
VEGF-A	188.85 ± 5.51	195.38 ± 8.25 ns	254.30 ± 15.39 ***^,##^

Data are presented as mean ± SEM (n = 12 per group). One-way ANOVA followed by Tukey’s post hoc test was used for statistical analysis. * *p* < 0.05, ** *p* < 0.01, *** *p* < 0.001 vs. control group; ^##^ *p* < 0.01, ^###^ *p* < 0.001 vs. oral ulcer group; ^ns^: not significant.

## Data Availability

The data presented in this study are available upon request from the corresponding author.
